# Development of a Raman-Based Method for the Diagnosis of People with Obstructive Sleep Apnea Syndrome: The Role of Lactic Acid

**DOI:** 10.3390/ijms26189095

**Published:** 2025-09-18

**Authors:** Luana Forleo, Silvia Picciolini, Alice Gualerzi, Elvia Battaglia, Elena Compalati, Paolo I. Banfi, Marzia Bedoni

**Affiliations:** 1 IRCCS Fondazione Don Carlo Gnocchi, 20148 Milan, Italy; lforleo@dongnocchi.it (L.F.); agualerzi@dongnocchi.it (A.G.); ebattaglia@dongnocchi.it (E.B.); ecompalati@dongnocchi.it (E.C.); pabanfi@dongnocchi.it (P.I.B.); mbedoni@dongnocchi.it (M.B.); 2 Department of Pathophysiology and Transplantation, University of Milan, 20122 Milan, Italy; 3 Sleep Center, Centro Diagnostico Italiano—C.D.I., 20147 Milan, Italy

**Keywords:** Raman spectroscopy, spectroscopy, saliva, obstructive sleep apnea syndrome, molecular biomarker, lactic acid, pulmonary rehabilitation

## Abstract

Obstructive sleep apnea syndrome (OSAS) is a respiratory disorder in middle-aged and elderly populations, marked by breathing interruptions during sleep. Diagnosis faces challenges due to the absence of specific biomarkers and increasing screening demands. This study aims to identify salivary biomarkers for the diagnosis and monitoring of OSAS using a simplified, rapid, and non-invasive method. Saliva from 51 OSAS patients and 34 healthy controls (CTR) was analyzed using Raman spectroscopy, which identified disease-specific biochemical fingerprints. Raman analysis revealed differences between the OSAS and CTR groups. The area under the curve of the lactic acid peak (920 cm^−1^) appeared higher in the OSAS group compared to the CTR group, suggesting potential diagnostic relevance. Significant correlations were found between biomolecular and clinical data, and a final linear model indicated that the lactate concentration significantly influenced the canonical variable. The Raman-based approach and the lactic acid peak represent a promising tool for OSAS diagnosis, monitoring, and supporting decisions in pulmonary rehabilitation. However, further investigation with a larger cohort is needed to clarify the observed discrepancies.

## 1. Introduction

Obstructive sleep apnea syndrome (OSAS) is a sleep-related breathing disorder that involves a decrease in or complete cessation of airflow and a continuous effort to breathe.

It affects about one billion people worldwide, more frequently men (24%) than women (9%), for which the prevalence increases especially during menopause [1,2,3]. OSAS occurs typically in middle-aged and elderly people, and the incidence increases with age or depending on anatomic factors, such as body mass index (BMI), craniofacial features, non-anatomic risk factors (ethnicity), additional factors (unhealthy habits such as alcohol consumption, smoking, and use of sedatives and/or hypnotics), and associated medical disorders [2,4,5,6]. The main features of OSAS comprise repetitive cessation of breathing (apnea) and decreased airflow caused by upper airway obstruction that results in blood oxygen desaturation (hypopnea).

The consequences of the disease involve sleep fragmentation, intermittent sympathetic activation, and muscular stress that leads to systemic oxidative stress and, consequently, cardiovascular (hypertension, heart failure, and coronary heart disease) and neurological complications [7,8,9] and diabetes; and, as reported by Cheng et al. in their meta-analysis, it could be associated with an overall increase in the incidence of cancer [10,11].

Moreover, daytime symptoms such as unrefreshing sleep, excessive sleepiness, fatigue, tiredness, lack of energy, and decreased attention and memory significantly affect the social life of OSAS patients and result in a reduction in work performance and an increase in car accidents or workplace incidents [12,13].

The gold standard for the diagnosis of OSAS is polysomnography (PSG), which determines the apnea and hypopnea index (AHI), i.e., the number of apnea and hypopnea events per hour of sleep. This index provides the assessment of the absence of the disease (AHI < 5/h) and the severity of OSAS from mild (AHI ≥ 5 and <15/h) to moderate (AHI ≥ 15 and ≤30/h) and severe disease (AHI > 30/h) [2,14]. Alternative diagnostic approaches, such as the use of screening tools, questionnaires, and the Home Sleep Apnea Test (HSAT), have also been considered. However, the current literature highlights several limitations of these tools, including lower accuracy compared with PSG, different physiological parameters collected, and the need for trained personnel to ensure correct sensor placement [14,15]. Concerning treatment, Continuous Positive Airway Pressure (CPAP) is the current gold standard for this pathology. However, evidence in the literature has shown that pulmonary rehabilitation can improve anamnestic dyspnea, body composition, and sleep-disordered breathing [16,17]. A recent prospective study proved that, following rehabilitation, health-related quality of life parameters had a significant improvement, demonstrating that rehabilitation can act as an adjunct to standard CPAP therapy to improve quality of life, deconditioning, and daytime sleepiness in patients with OSAS [16]. Still, no biomarker exists to objectively prove rehabilitation effectiveness.

Currently, the expected increase in OSAS diagnoses due to the growing prevalence and severity of obesity, as well as the rising number of mandatory screenings (i.e., for a driver’s license), is a significant issue for healthcare systems in terms of patient management [18], making OSAS both a clinical and social challenge. Indeed, although many attempts to identify OSA biomarkers have been made over the past two decades, the results have often lacked reproducibility, and the proposed candidates have generally not fulfilled the criteria required for clinical implementation [19,20]. All these observations, along with the lack of specific biomarkers associated with the condition, emphasize the growing clinical need to diagnose and manage OSAS by simplified tests, based on the analysis of easily accessible, non-invasive, and painless biofluids through effective, sensitive, and affordable methods [18].

In this context, our study aims to evaluate the effectiveness of Raman Spectroscopy (RS) on salivary samples, as a fast, sensitive, innovative, and accurate biophotonic method for the diagnosis, monitoring, and personalized therapy of OSAS patients.

RS is a vibrational spectroscopy technique, which has been valued as extremely promising in chemical biology research [21], diagnostics, and biomedical analysis thanks to its rapidity, label-free nature, high sensitivity, specificity, and the minimal or absent sample preparation required [22,23,24]. RS applied on biological samples provides a characteristic spectrum, a fingerprint, that represents a combination of concentrations, interactions, modifications, and presence of physiological and pathological biomolecules, reflecting the complex biochemical composition [25,26].

Thanks to its unique properties, RS can be employed to characterize salivary samples by identifying the specific salivary fingerprints associated with various physiopathological conditions. RS is here applied to biochemically profile salivary samples and to identify a spectroscopic biomarker of OSAS. Indeed, saliva is a highly informative biofluid, composed of 99% water, and contains about 2300 proteins, enzymes, inorganic salts, and hormones shared with blood [27,28]. Moreover, it is an easily accessible and low-cost biofluid. However, the characteristics of saliva, such as the limited concentrations of analytes, require the use of advanced technologies for an accurate evaluation of the potential markers to overcome the challenges associated with the collection, analysis, and interpretation of salivary data [29,30]. To date, only a limited number of studies have investigated saliva for the identification of biomarkers related to OSAS [31,32,33]. However, the extensive literature on the use of RS on salivary samples for the diagnosis and monitoring of various diseases, such as neurological, respiratory, tumoral, or infectious conditions [34,35,36,37,38,39], confirmed the validity and potential of the methodology. For example, respiratory diseases, including COVID-19 and chronic obstructive pulmonary disease (COPD), have been investigated using RS, revealing the distinct molecular fingerprints associated with inflammation and infection [27,40,41]. The intended outcomes of our study are to identify specific salivary molecular Raman fingerprints associated with OSAS patients and to assess the diagnostic accuracy of the method in distinguishing OSAS patients from healthy individuals. Moreover, considering the demonstrated relationship between nocturnal hypoxia and lactate levels in sleep-related breathing disorders [42], an in-depth analysis on Raman spectra is performed to investigate if the presence of lactic acid affects the spectral differences among experimental groups, and to explore correlations between Raman and clinical data. These analyses can help to assess the diagnostic potential of RS of saliva for OSAS patients, and to demonstrate the feasibility of saliva as a practical and accessible biofluid for routine screening. The originality of the proposed work lies on the use of such a sensitive technology on the saliva of individuals with OSAS, as it provides an ideal solution for rapid, easy, non-invasive, and painless diagnosis and cost-effective, practical management [43].

## 2. Results

### 2.1. Participants Characterization

Eighty-five participants were recruited, and information regarding demographic and clinical parameters was collected, as reported in Table 1. No statistical difference was observed for age and sex between the two groups (Nonparametric Mann–Whitney test).

### 2.2. Raman Fingerprint of Salivary Samples

**Figure 1:**
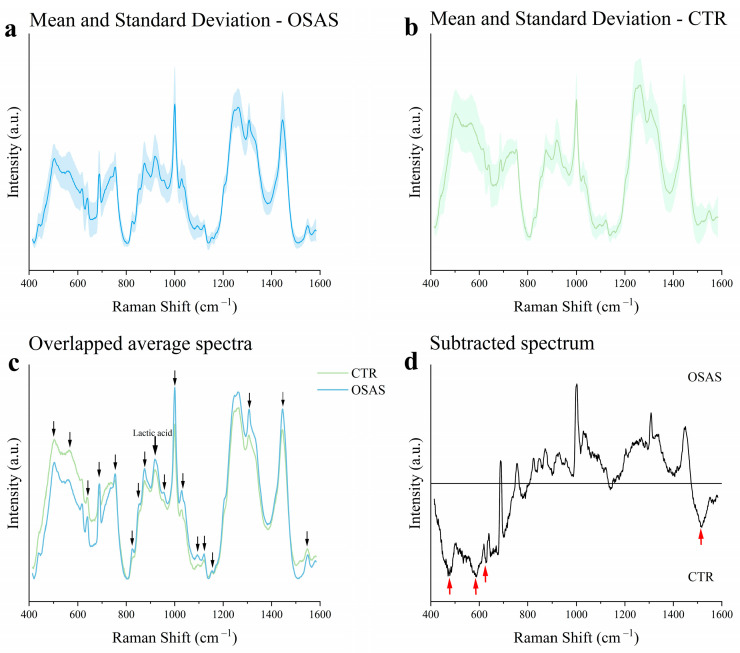
Raman analysis. (**a**,**b**) Mean Raman spectra and standard deviations of the considered groups. (**c**) Overlapped average spectra and spectral regions identification with the greatest differentiation between groups (indicated by the black arrows); a bold black arrow indicates the Lactic acid peak (920 cm ^−1^). (**d**) Subtracted spectrum between OSAS and CTR averaged spectra; red arrows indicate peaks more prominent in the CTR group.

The Raman analysis of saliva was performed by optimizing the analytical protocol developed by our group [40]. During the acquisition, spectra with no visible peak at 1000 cm^−1^ were discarded. Ten spectra per sample were successfully acquired for all samples in random spots of the saliva drop. The mean spectrum for each subject was calculated. Then, the average spectra for the OSAS and CTR groups were computed (Figure 1a,b). Both OSAS and CTR average spectra exhibited a consistent shape and a good signal-to-noise ratio. Standard Deviation (SD) appeared more pronounced in the CTR group compared to the OSAS group. The overlap of the mean OSAS and CTR spectra showed remarkable differences in the salivary fingerprint of the two groups (Figure 1c). Differences in several Raman peaks were observed, and each of them was assigned to specific chemical bonds based on literature data [25]. The assignments are reported in Table 2, highlighting notable peaks at 920 cm^−1^ (Lactic acid), 957 cm^−1^ (Carotenoids), 1030 cm^−1^ (Collagen), and 1444 cm^−1^ (Cholesterol), which are higher in the OSAS group than in the CTR group. Other peaks that differ in the relative intensity between the two groups referred to proteins (618 cm^−1^, 640 cm^−1^, 755 cm^−1^, 853 cm^−1^, 1003 cm^−1^, 1308 cm^−1^, 1548 cm^−1^), lipids (875 cm^−1^, 1095 cm^−1^), nucleic acids (825 cm^−1^, 1095 cm^−1^, 1120 cm^−1^), and carbohydrates (1153 cm^−1^). Specifically, some protein peaks (618 cm^−1^, 640 cm^−1^, 1548 cm^−1^) were more intense in CTR than in the OSAS group (Figure 1c). The subtraction of the CTR mean spectrum from the OSAS average spectrum showed an abundance of saccharides (477 cm^−1^), phosphate of hydroxyapatite (589 cm^−1^), glycerol (630 cm^−1^), and β-carotene (1517 cm^−1^) in the CTR group compared to the OSAS, as shown in Figure 1d.

### 2.3. Multivariate Statistical Analysis

**Figure 2:**
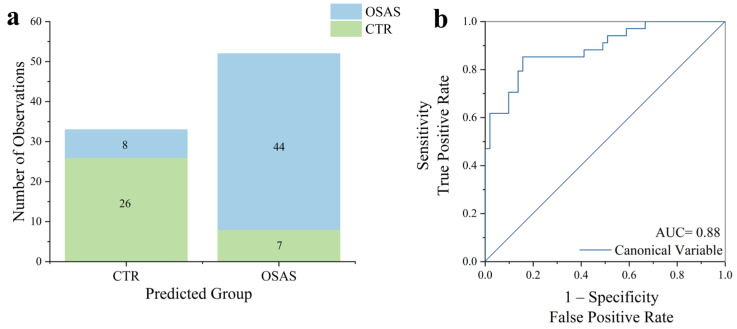
Linear Discriminant Analysis (LDA) results. (**a**) Histogram showing the number of observations obtained through the classification model for each group. (**b**) Receiver Operating Characteristic (ROC) curve calculated for the determination of the goodness of the proposed classification model based on the Raman analysis of saliva from OSAS and CTR subjects.

To verify if the observed differences could lead to the creation of a classification model able to discriminate the Raman signals collected from OSAS and CTR subjects, Principal Component Analysis—Linear Discriminant Analysis (PCA-LDA) was performed and validated with a Leave-One-Out Cross Validation (LOOCV). After computing the average spectrum for each patient, the resulting data created a matrix of 85 × 967 points (Nsamples × Nfeatures). Six PCs were selected to minimize the error rate during the LOOCV in the LDA validation. Given the two groups in the dataset, the LDA computed one canonical variable, and the cross-validation yielded an overall error rate of 18.63%. The error rates of the single groups were as follows: 13.73% for OSAS and 23.73% for CTR. The obtained results allowed for the determination of the model’s accuracy, precision, sensitivity, and specificity, as well as its ability to correctly discriminate between the spectra of the two different groups under analysis. The values of these metrics were, respectively, 82.35%, 86.27%, 84.62%, and 76.47%. The histogram resulting from the classification is shown in Figure 2a. Additionally, to assess the goodness of the classification model, the Receiver Operating Characteristic (ROC) curve was also performed using the canonical variable (Figure 2b). An area under the curve (AUC) value of 0.88 and an asymptotic probability <0.0001 were obtained, confirming the strong ability of the model to accurately discriminate between subjects across groups.

### 2.4. Analysis of the Lactic Acid Raman Peak

**Figure 3:**
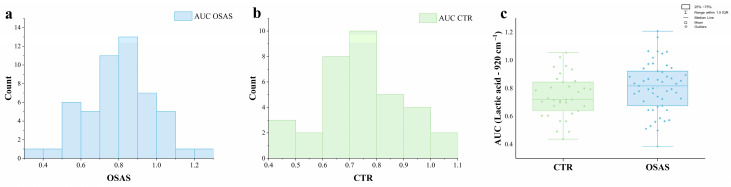
Area Under the Curve (AUC) of the lactic acid peak. (**a**,**b**) Distribution of AUC for the Lactic acid in OSAS and CTR groups. (**c**) Box plot reporting the AUC scores obtained for CTR and OSAS. Mean values were proved not to be statistically different when compared using two-sample *t*-test (*p* < 0.05).

**Figure 4:**
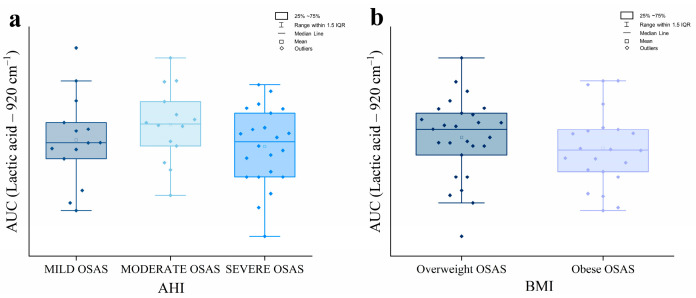
Box plots related to the Area Under the Curve (AUC) of the Lactic acid peak in OSAS grouped by Apnea and Hypopnea Index (AHI) (**a**) and Body Mass Index (BMI) (**b**).

To verify the differences in the spectral contribution of lactic acid in the salivary fingerprint of OSAS and CTR, the distributions of AUC values related to the lactic acid peak (920 cm^−1^) were measured. As shown in Figure 3a,b, the results demonstrate the typical Gaussian distribution for both experimental groups, confirmed by the Shapiro–Wilk test. The mean AUC values obtained were M = 0.74, SD *=* 0.15 for the CTR group and M = 0.80, SD *=* 0.17 for the OSAS group. A parametric two-sample t-test was then used to determine if there were statistically significant differences between the groups’ AUC values. At the 0.05 significance level, no significant difference was observed (*p* = 0.07; Figure 3c); however, a higher trend in AUC was noted in the OSAS group compared to the CTR. To examine whether the lactic acid AUC varied with the severity of OSAS and degree of obesity, salivary samples were initially divided into three groups based on AHI (mild, moderate, and severe OSAS) and into two groups based on the BMI (overweight, 25–29.99 kg/m^2^; obese, > 30 kg/m^2^) according to the WHO guidelines. The lactic acid AUC was then compared between groups using the ANOVA One Way test and the two-sample *t*-test for severity and obesity degree, respectively. As shown in Figure 4, lactic acid AUC had not shown differences either between OSAS grouped by AHI (*p =* 0.29) (Figure 4a) or between OSAS grouped by BMI (*p =* 0.34) (Figure 4b), a finding that may be influenced by the limited subgroup sample size and the restricted profiling of CTR group; further investigation will allow us to confirm or disprove the here-observed trend.

### 2.5. Quantification of Target Molecules in Salivary Samples

**Figure 5:**
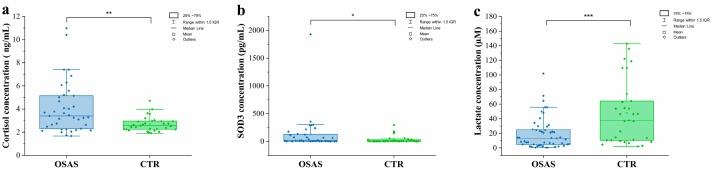
Box plots showing the distributions of the concentrations of cortisol (**a**), Superoxide Dismutase 3 (SOD3) (**b**), and lactate (**c**) obtained using Enzyme-Linked ImmunoSorbent Assay (ELISA) and colorimetric assays on the salivary samples of OSAS and CTR subjects. * *p* < 0.05, ** *p* < 0.01, and *** *p* < 0.001.

Fluorimetric and Enzyme-Linked Immunosorbent Assay (ELISA) assays were performed to characterize the biomolecular composition of saliva using conventional techniques. The data from these analyses were crucial for validating the spectral observations obtained using the innovative technology of RS. First, cortisol concentration was tested, as it is a well-known stress biomarker present in saliva. As shown in Figure 5a, the results from the immuno-quantification of cortisol demonstrate that the concentration levels of this biomarker were different in the salivary samples of the OSAS and CTR groups, with higher levels in OSAS patients (Mann–Whitney test, *p* < 0.05). The results of the ELISA of SOD3 show significant differences between the experimental groups (Figure 5b, Mann–Whitney test, *p* = 0.042): a positive trend was observed in the OSAS group compared to the CTR. Regarding the fluorimetric assays used to quantify the lactate, the results reveal a higher salivary concentration in the CTR than in the OSAS group (Figure 5c). The median concentration of salivary lactate was 13.23 µM (*IQR* = 20.51) for OSAS and 37.76 µM (*IQR* = 54.04) for CTR. The Mann–Whitney test demonstrated a statistically significant difference between the two groups (*p* < 0.001). Surprisingly, although the Raman spectrum and the AUC corresponding to the lactic acid peak showed greater intensity in the OSAS group compared to the CTR, the quantification of lactate showed an opposite result, which warrants more in-depth investigation and validation in a larger cohort.

### 2.6. Correlation Analysis

**Figure 6:**
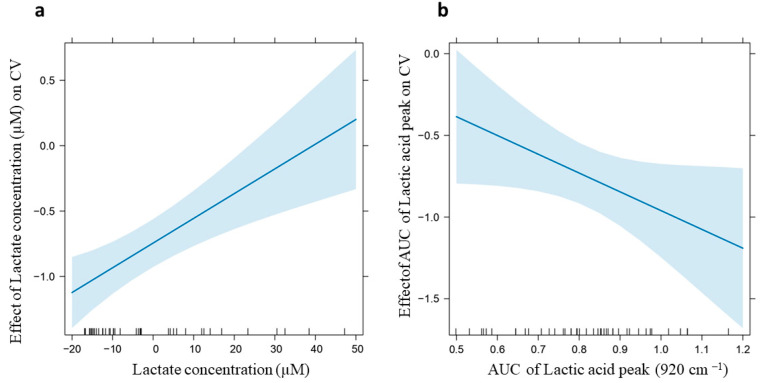
The Linear Model (LM) representation. (**a**) Effect of lactate concentration (*x*-axis) on CV (*y*-axis) in patients with OSAS. (**b**) Effect of Area Under the Curve (AUC) of lactic acid (*x*-axis) on CV (*y*-axis) in patients with OSAS. The blue lines represent the linear trend, while the light-blue areas indicate the confidence intervals.

To assess the presence of potential correlations between experimental and clinical data related to OSAS patients, Spearman correlation analysis and two different Linear Models (LMs) were performed. The results of the Spearman correlation (Figure S1) demonstrate that, in our cohort, there are no correlations between the Raman-based classification model (canonical variable score) and disease severity (expressed as AHI), Epworth Sleepiness Scale (ESS) score, and the smoking habit. However, a significant positive Spearman correlation was observed between the canonical variable (CV) values obtained from the Raman classification model and the lactate concentration of OSAS patients, with a correlation coefficient of *r =* 0.43 and a *p* = 0.002. Furthermore, a borderline statistically significant correlation was found between CV and the AUC of the lactic acid peak at 920 cm^−1^ with a correlation coefficient of *r =* −0.28 and *p* = 0.051. Moreover, the AUC of the lactic acid peak showed a negative correlation with BMI (correlation coefficient *r* = −0.33, *p* < 0.05). Based on the Spearman correlation between CV and lactate concentration, an LM including a quadratic component for lactate was subsequently implemented to identify and remove potential outliers and influent data. Subsequently, a simple LM was performed, also considering the near-significant correlation between CV and the AUC of the lactic acid peak. The results of the LM indicate that only the lactate concentration has a statistically significant positive effect on the CV (*p* < 0.001, coefficient *β*1 = 0.019; Figure 6a). As we observed that negative CV values are mainly related to OSAS patients, this positive correlation suggests that the lactate concentration, obtained using a fluorimetric assay, is higher in the saliva of CTR subjects compared to OSAS. In contrast, the AUC of the lactic acid peak shows a negative trend, although this effect is not statistically significant (*p* = 0.05, coefficient *β*2 = −1.15; Figure 6b), which may suggest an increased AUC in patients with OSAS compared to CTR. It should be noted that the AUC values obtained for the lactic acid peak revealed no correlation with the lactic acid concentration obtained using the fluorimetric assay. This might be explained by the observation that the peak at 920 cm^−1^ is the main and the only detectable peak of lactic acid in saliva, but the pure molecule is characterized by a Raman spectrum, with several peaks that might be hindered by other salivary components (Figure S2).

## 3. Discussion

OSAS is a chronic, debilitating syndrome that requires expensive diagnostic tools. Unfortunately, many people with OSAS experience delayed diagnoses because symptoms are underestimated and there are no accessible non-invasive screening options. Nonetheless, the management of OSAS, ranging from diagnosis to treatment, currently represents a challenge for healthcare systems, also due to the expected increase in OSAS diagnoses associated with the growing prevalence and severity of obesity. The present study proposes a novel, reliable screening method for OSAS through the non-invasive analysis of saliva by Raman spectroscopy. The salivary test proved to be effective in the diagnosis of OSAS, with a very good diagnostic accuracy of 88%. The use of saliva in diagnostics is now emerging as a game changer in multiple pathologies, especially chronic diseases that require frequent and periodical monitoring, thanks to its remarkable advantages compared to other liquid biopsies: non-invasiveness, cost-effectiveness, easy laboratory storage, and handling. As a demonstration of its great potential, the number of commercially available salivary tests and saliva collection kits is exponentially increasing, as well as the number of publications on its use in diagnostics [46]. However, it has to be noted that the use of saliva for diagnostic testing still faces a technological challenge due to the limited concentrations of analytes compared to other commonly used fluids like blood. In this context, the use of RS has been recently proven to be effective in overcoming the detection limits of other investigation methods when applied to saliva, demonstrating its potential for the diagnosis and monitoring of complex chronic disorders [27,40,47,48]. In addition, RS has the significant advantage of providing a spectral fingerprint of the biological sample that represents a measurable biomarker and contains multifaceted information coming from all the biomolecular components, such as lactic acid, carotenoids within the analyzed specimen [33,49]. This makes RS one of the current emerging omic approaches [50]. In the present work, we demonstrate that the salivary spectral fingerprint of OSAS patients has significant differences compared to CTR subjects of comparable age. Specifically, we were able to identify several peaks that can be attributed to altered oxidative status, like Carotenoids (957 cm^−1^), which represent an important antioxidant moiety and are more prominent in OSAS patients compared to controls. Such spectral difference may be due to carotenoid function and accumulation during a long period of hypoxia. This data about the altered oxidative stress status of OSA is also in agreement with the analysis of SOD3 levels in saliva that showed an overexpression of this antioxidant in OSAS, capable of converting superoxide anions into hydrogen peroxide and oxygen to protect tissues from damage caused by oxidative stress [51]. The molecular analyses of saliva showed higher levels of cortisol in the saliva of OSAS patients compared to controls, possibly due to greater chronic stress in patients. Indeed, as reported by Imani et al., when stress factors persist continuously, the adaptive capacity of the hypothalamic–pituitary–adrenocortical axis (HPAa) (responsible for cortisol regulation) decreases, and cortisol secretion may remain either consistently high or consistently low [52]. On the other hand, the Raman analysis of saliva has also found Collagen (1030 cm^−1^)- and Cholesterol (1444 cm^−1^)-related peaks to be more prominent in OSAS spectra, possibly reflecting the involvement of these molecules in the remodeling and fibrosis of the upper airways, which is common in OSAS and is also associated with the increased cardiovascular risk of these patients [53,54]. Interestingly, we report an increased trend regarding the AUC of the lactate peak (920 cm^−1^) in people with OSAS compared to controls. Despite not reaching statistical significance, probably due to the limited sample size of CTR group, such observation warrants particular attention, as there is evidence in the literature which has highlighted the important role of lactic acid as a biomarker for OSAS, even in comparison to other biomarkers such as uric acid. The greater anaerobic degradation of glucose during the night, due to the hypoxic oxidative stress state of OSAS, triggers the action of lactate dehydrogenase and the synthesis of lactate, which accumulates and causes hyperlactatemia in the plasma of patients with OSAS [55]. These findings also align with our understanding of OSAS pathophysiology, where increased respiratory effort during sleep may lead to enhanced muscle activity and subsequent lactate production [56]. However, when we analyzed the trend in the lactate concentration between OSAS and CTR, the results do not seem to reflect the data obtained through the Raman analysis and the evaluation of the AUC of the lactic acid peak. We speculate that the small sample size, together with the limited profilation of the CTR group, may have influenced the results obtained. Additionally, the correlation results are considered controversial, and require further investigation for a more comprehensive interpretation. We could hypothesize that the salivary lactate concentration may be influenced by different factors such as the diet, hydration, the time of saliva sample collection, or by technical variables of the conventional methods [57,58]. Although the present results require further validation on a wider cohort, the reported data confirm lactic acid as a key player in the biochemical changes observed in OSAS saliva, and, based on the multivariate analysis of Raman data, we suggest that the spectral signature of lactic acid is a potential biomarker for the diagnosis of OSAS. However, we have to mention that the discrepancy between the Raman spectroscopy results and lactate concentration measurements in the fluorimetric assay requires further investigation to understand the underlying mechanisms and reasons for the observed differences. As shown, the Raman peaks related to lactic acid are not limited to the peak at 920 cm^−1^, but consist of several other peaks that might be hindered by the presence of other salivary components. Biological spectra contain many overlapping peaks, making the assignment of weak metabolite bands difficult in the presence of dominant protein signals. Mucins and other proteins in saliva contribute intense amide bands that dominate vibrational spectra and may obscure the signals of lower-abundance metabolites [59,60]. Moreover, ELISA and fluorimetric conventional assays require complex operational steps, resulting in less efficient methods that are challenging to reproduce reliably [61].

## 4. Materials and Methods

### 4.1. Study Design

In this descriptive cross-sectional study, a cohort of 85 participants was recruited at the Sleep Center, Unit of Cardiopulmonary Rehabilitation at IRCCS Fondazione Don Carlo Gnocchi ONLUS (Milan, Italy) between September 2022 and November 2024 after providing written informed consent (Protocol ID: 03_23/02/2022; approved by the Ethical Committee of Fondazione Don Carlo Gnocchi on 23 February 2022). Enrolled participants comprised 51 patients with a diagnosis of obstructive sleep apnea syndrome (OSAS; ages 25–82; 22 females, 29 males) and 34 healthy controls (CTR; ages 31–88; 19 females, 15 males). A statistical power of 72% was calculated through a post hoc analysis using Gpower software (ver. 3.1.9.7), setting a medium effect size (*f* = 0.5), an *α* level of 0.05, and the sample sizes of the considered groups. A non-parametric Mann–Whitney test was performed to verify that the age distribution was not significantly different between the two experimental groups (significance level with *p* < 0.05). Exclusion criteria for both groups were age < 18 years, the coexistence of pulmonary diseases (i.e., COPD and asthma), cancer, gingivitis, periodontal diseases, general gum bleeding, oral bacterial and fungal infections, recent dental operations, and other significant comorbidities, including cardiovascular and kidney diseases. Regarding OSAS patients, only subjects with a diagnosis based on PSG and AHI disease staging were recruited within the same timeframe. Information regarding smoking habits (non-smoker, smoker, or former smoker), BMI, ESS, and treatments were also recorded (Table S1). The primary variables that this study aimed to measure was the Raman fingerprint related to salivary samples from OSAS patients and healthy subjects in order to evaluate the effectiveness of RS as a fast, sensitive, innovative, and accurate method for the diagnosis, clinical monitoring, and personalized treatment of OSAS patients. As secondary variables, the AUC of the peak related to lactic acid, and the values of the correlation studies between the Raman fluorimetric assays and clinical data were calculated.

### 4.2. Saliva Collection and Processing

Salivary samples were collected using Salivette^®^ (Sarstedt AG & CO, Numbrecht, Germany) following the manufacturer’s instructions and a previously published protocol optimized for the clinical setting [40]. Briefly, a Salivette^®^ cotton swab was chewed for one minute to stimulate salivation and guarantee the collection of an adequate saliva volume. The collection procedure was performed at least one hour after the last meal and teeth-brushing. Then, the Salivette^®^ was frozen at −20 °C until processing by centrifugation at 1000× *g* for 2 min at +4 °C.

### 4.3. Raman Analysis

Raman analysis was conducted using the Raman micro-spectroscope LabRAM Aramis (Horiba Jobin Yvon S.A.S, Lille, France) equipped with a 785 nm laser source. A drop of 3 µL of saliva was deposited on an aluminum slide (Platypus Technologies, LLC, Madison, WI 53711, USA) and dried at room temperature for 10–15 min. Raman acquisitions were conducted using a 50 × objective (Olympus, Japan Tokyo 192-8507), ranking at the border of the drop in the so-called “coffee ring”. Acquisition parameters were set as follows: 600 grooves/mm diffraction grating, 200 µm entrance slit, 400 µm hole, spectral range 400–1600 cm^−1^, and acquisition time of 30 s. About 10 spectra for each subject were acquired. The Raman calibration was performed daily using as a reference the peak at 520.7 cm^−1^ of a silicon substrate.

### 4.4. ELISA and Colorimetric Assays

Concentrations of Cortisol, SOD3, and Lactate in salivary samples were evaluated using an ELISA and colorimetric assay. For the quantitative determination of salivary cortisol concentration, a sensitive commercial enzyme immunoassay able to detect cortisol in the range of 0.2–10 ng/mL (R&D Systems, Biotechne^®^, Minneapolis, MN, USA) (detection sensitivity 0.111 ng/mL) was employed. Saliva was diluted fivefold, and 50 µL was mixed with 200 µL of diluent according to the manufacturer’s protocol [62]. Cortisol concentrations were reported in ng/mL, and a cortisol standard curve was included on each assay plate. A fluorimetric assay for the determination of salivary lactate levels (Cell Biolabs, Inc., San Diego, CA, USA) (detection sensitivity of 1.5 µM) was utilized as previously reported [63]. The procedure involved the oxidation of lactate by lactate oxidase into pyruvate and hydrogen peroxide, which is then detected with a fluorometric probe. Horseradish peroxidase catalyzed the reaction between the probe and hydrogen peroxide, which bound in a 1:1 ratio. Undiluted salivary samples and standards were incubated for 30–45 min. Each sample was compared to a known concentration of lactate standard. For SOD3 quantification, an ELISA kit already validated for salivary samples (CUSABIO^®^, Biotech Co., Ltd., Wuhan, China) (detection sensitivity of 1.95 pg/mL) was also used; the kit had a detection range of 7.8 pg/mL–500 pg/mL. Samples were centrifuged for 10 min at 4000× *g* at +4 °C to remove particulates and assayed immediately following the manufacturer’s instructions. In total, 100 μL of undiluted saliva was used. For the determination of absorbance values related to the 3 kits, the CLARIOstar Absorbance microplate reader (BMG LABTECH, Ortenberg, Germany) with a filter set at 450 nm was utilized. To calculate the concentration of salivary levels of cortisol, lactate, and SOD3, a standard curve was performed.

### 4.5. Statistical Analysis

Raman data were pre-processed using LabSpec6 software (ver. 6.4.3.35; Horiba Jobin Yvon S.A.S, Lille, France) to standardize the dataset. Firstly, a third-degree polynomial baseline was subtracted from the spectra to reduce general background intensity deriving from fluorescence and photoluminescence. Then, a reference peak (1000 cm^−1^) was used to align all the spectra and, finally, the data were saved with a final resolution of 1.21 cm^−1^ per step and 969 points for a single spectrum. Ten spectra per subject were averaged to obtain a ratio of 1:1 between the Raman spectrum and the patient using OriginPro2023b software. To reduce the data dimensionality, preserving important information from all spectra, PCA was applied to the patients’ average spectra of both groups and used to extract the Principal Components (PCs) that best describe the variance between the data [64]. In total, 6 PCs were then used to perform LDA and develop a classification model to distinguish the differences in the Raman spectra based on the resulting CV using the LOOCV. The accuracy of the method was calculated, and the CV scores were used to compute the ROC curve for the evaluation of the diagnostic accuracy. Moreover, the AUC of the lactic acid peak (920 cm^−1^) was calculated for all spectra, integrating the Raman intensities in the range between 910.75 cm^−1^ and 930.13 cm^−1^ using OriginPro2023b. Descriptive statistics were used to obtain the mean value for each group; the statistical difference between the AUC distributions was tested with a two-sample *T*-test after verifying the normality of all the data using the Shapiro–Wilk test. To analyze data derived from the ELISA and fluorimetric assays, a normality test (Shapiro–Wilk test) was performed to verify the distribution. Assuming a non-normal distribution, a non-parametric test (Mann–Whitney test) was applied to all data to evaluate the differences between the concentrations in the experimental groups. Differences were considered statistically significant at a *p* < 0.05. Statistical analysis was conducted using OriginPro 2023 b software.

### 4.6. Correlation Analysis

A descriptive statistical analysis to evaluate the presence of significant correlations was performed. Specifically, Raman data (CV and the AUC of the lactic acid peak) and the fluorimetric assay results (lactate concentration) were correlated with clinical data such as the BMI and AHI by Spearman analysis (Figure S1). Since Spearman correlation analyses revealed interesting relationships between the CV and lactate concentration, an LM with a quadratic term for lactate was applied. Absence of multicollinearity was verified, and outliers were removed. The homoscedasticity and normality of residuals were then checked. After removing the outliers and influential values, another simple LM was performed, including the near-significant correlation between CV and AUC of lactic acid peak, showing the effects of lactate concentration and the AUC on CV. Correlation results were considered statistically relevant for *p* < 0.05. These analyses were performed using RStudio software (ver. 4.5.1). Additional information is available in the Supplementary Materials.

## 5. Conclusions

In conclusion, the herein reported data demonstrate that Raman spectroscopy applied on salivary samples represents a powerful investigation tool that can shed light on the pathophysiology of OSAS, underlying a relevant role of lactate concentration in disease onset. Our results indicate that the spectral signature of lactic acid could be a potential biomarker for OSAS and that the proposed approach can provide clinicians with a new measurable tool for the diagnosis of OSAS, the monitoring of its progression, and potentially the evaluation of the efficacy of pulmonary rehabilitation treatments, reducing costs and increasing accessibility and quality of care. This latter application of saliva Raman testing should be further verified, but it could open the way to new personalized therapies and rehabilitation treatments for people with OSAS.

## Figures and Tables

**Table 1:** Main characteristics of the recruited participants. A summary of the demographic (age and sex) and clinical (AHI) features of the recruited participants is reported. The Mann–Whitney test was used to verify the age and sex match between the groups (significance level for *p* < 0.05).

	AGE	SEX		AHI (events/h)		
	**Median** (*IQR*)	**M** (%)	**F** (%)	**5–15** (%)	**15–30** (%)	**>30** (%)
**OSAS** (*n* = 51)	66 (14)	29 (57%)	22 (43%)	14 (27%)	14 (27%)	23 (45%)
**CTR** (*n* = 34)	61.5 (21)	15 (44%)	19 (56%)			
***p-value***	0.58	0.43				

*IQR*: Interquartile Range; AHI: Apnea and Hypopnea Index; OSAS: Obstructive Sleep Apnea Syndrome; CTR: Healthy Controls.

**Table 2:** Attribution of the main peaks obtained from the Raman analysis of salivary samples from both OSAS and CTR groups [25,44,45].

Raman Shift (cm^−1^)	Attribution				
	Nucleotides	Proteins	Lipids	Carbohydrates	Pigments
**505**	Methoxy group				
**589**				Glycerol	
**618**		C-C twisting of proteins			
**630**				Glycerol	
**640**		C-S stretching and C-C twisting of Tyrosine			
**755**		Tryptophan			
**825**	Phosphodiester bond				
**853**		Tyrosine and Proline		Glycogen	
**875**			Phospholipids (Phosphatidylcholine, sphingomyelin)		
**920**		C-C stretch of proline ring		Glucose/ Lactic acid	
**957**					Carotenoids
**1003**		Phenylalanine			
**1030**		Phenylalanine of collagen			
**1095**	Phosphodioxy group		C-N		
**1120**	The strong C-O band of ribose				
**1153**				Carbohydrates peak	
**1250**	Aluminum substrate band				
**1308**		C-N asymmetric stretching in asymmetric aromatic amines			
**1444**			Cholesterol band, fatty acids		
**1548**		Tryptophan			

## Data Availability

All data and analysis are available within the manuscript or upon request to the corresponding author.

## Supplementary Materials

The following supporting information can be downloaded at: https://www.mdpi.com/article/10.3390/ijms26189095/s1.

## Abbreviations

The following abbreviations are used in this manuscript:

AHI	Apnea and Hypopnea Index
AUC	Area Under the Curve
BMI	Body Mass Index
CPAP	Continuous Positive Airway Pressure
CTR	Healthy controls
CV	Canonical Variable
ELISA	Enzyme-Linked ImmunoSorbent Assay
ESS	Epworth Sleepiness Scale
HPAa	Hypothalamic–Pituitary–Adrenocortical axis
HSAT	Home Sleep Apnea Test
LDA	Linear Discriminant Analysis
LM	Linear Model
LOOVC	Leave One Out Cross Validation
OSAS	Obstructive sleep apnea syndrome
PCA	Principal Component Analysis
PSG	Polysomnography
RS	Raman spectroscopy
SOD3	Superoxide Dismutase 3
