# Supplementary material for: Development of a Raman-Based Method for the Diagnosis of People with Obstructive Sleep Apnea Syndrome: The Role of Lactic Acid
Source: Int J Mol Sci. 2025 Sep 18;26(18):9095. doi: 10.3390/ijms26189095 (PMC12470120; doi:10.3390/ijms26189095)
Supplement: Supplementary file 1 [file ijms-26-09095-s001.zip › ijms-3833539-supplementary.pdf]

SUPPLEMENTARY MATERIALS

Development of a Raman-based method for the diagnosis of people with obstructive sleep apnea syndrome: the role of lactic acid

Luana Forleo[1,2], Silvia Picciolini[1] *, Alice Gualerzi[1], Elvia Battaglia[1,3], Elena Compalati[1], Paolo I. Banfi[1], Marzia Bedoni[1]

[1] IRCCS Fondazione Don Carlo Gnocchi, 20148 Milan, Italy
[2] Department of Pathophysiology and Transplantation, University of Milan, 20122 Milan, Italy
[3] Sleep Center, Centro Diagnostico Italiano—C.D.I., 20147 Milan, Italy

Corresponding Author:
* Silvia Picciolini
Via Capecelatro, 66 - 20148 Milan, Italy
+39 02 4030 8533
spicciolini@dongnocchi.it

2. METHODS

2.1 STUDY DESIGN

SAMPLE ID	OSA SEX	OSA AGE	BMI	ESS	SMOKE	THERAPIES	AHI
ID1	F	40	25	5	Yes	No CPAP	13
ID2	M	67	29	4	Ex	CPAP	40
ID3	F	77	32	2	No	CPAP	54
ID4	M	46	30	6	No	CPAP	40
ID5	F	64	28	2	Ex	CPAP	56
ID6	F	50	26	3	Ex	CPAP	31
ID7	F	60	28	18	No	CPAP	8
ID8	F	71	31	4	No	CPAP	7
ID9	F	79	29	2	No	CPAP	29
ID10	M	69	34	10	Ex	CPAP	12
ID11	M	61	29	4	Yes	CPAP	27
ID12	F	60	44	6	Yes	CPAP	11
ID13	M	60	36	10	Ex	CPAP	14
ID14	M	76	26	12	Yes	CPAP	30
ID15	M	69	27	6	No	CPAP	25
ID16	M	73	26	12	No	CPAP	35
ID17	F	76	28	12	No	CPAP	7
ID18	M	60	28	9	No	CPAP	10
ID19	F	82	36	10	No	CPAP	42
ID20	M	40	30	12	Ex	CPAP	49
ID21	F	69	25	4	No	CPAP	10
ID22	M	59	28	6	No	CPAP	29
ID23	F	69	34	14	No	CPAP	45
ID24	M	64	30	10	Ex	Other therapy	30
ID25	M	64	28	18	Ex	CPAP	22
ID26	F	74	9	10	No	CPAP	17
ID27	F	66	27	14	Ex	CPAP	70
ID28	F	47	30	16	Ex	CPAP	23
ID29	F	68	28	10	No	CPAP	20
ID30	F	63	26	18	No	CPAP	15
ID31	M	67	27	9	No	CPAP	11
ID32	M	67	39	6	No	CPAP	98
ID33	M	32	42	14	Yes	CPAP	48
ID34	M	73	44	12	No	CPAP	17
ID35	M	71	26	8	Ex	CPAP	46
ID36	M	53	35	4	Ex	CPAP	15
ID37	M	75	26	2	Ex	CPAP	41
ID38	M	70	28	10	No	CPAP	74
ID39	M	46	36	8	No	CPAP	6
ID40	F	50	37	12	Ex	CPAP	46
ID41	M	70	41	14	Yes	CPAP	76

**ID42**	M	41	40	12	No	CPAP	48
**ID43**	F	57	25	8	No	CPAP	29
**ID44**	M	82	26	2	No	CPAP	46
**ID45**	M	64	26	4	Yes	CPAP	32
**ID46**	M	59	30	14	No	CPAP	44
**ID47**	M	49	42	16	No	CPAP	69
**ID48**	F	70	24	10	No	CPAP	6
**ID49**	F	66	29	8	No	CPAP	16
**ID50**	M	25	29	12	Ex	CPAP	26
**ID51**	F	72	33	3	No	CPAP	52

Table S1. Patients information. Body Mass Index (BMI); Continuous Positive Airway Pressure (CPAP); Epworth Sleepiness Scale (ESS); Apnea and Hypopnea Index (AHI).

2.6 CORRELATION ANALYSIS

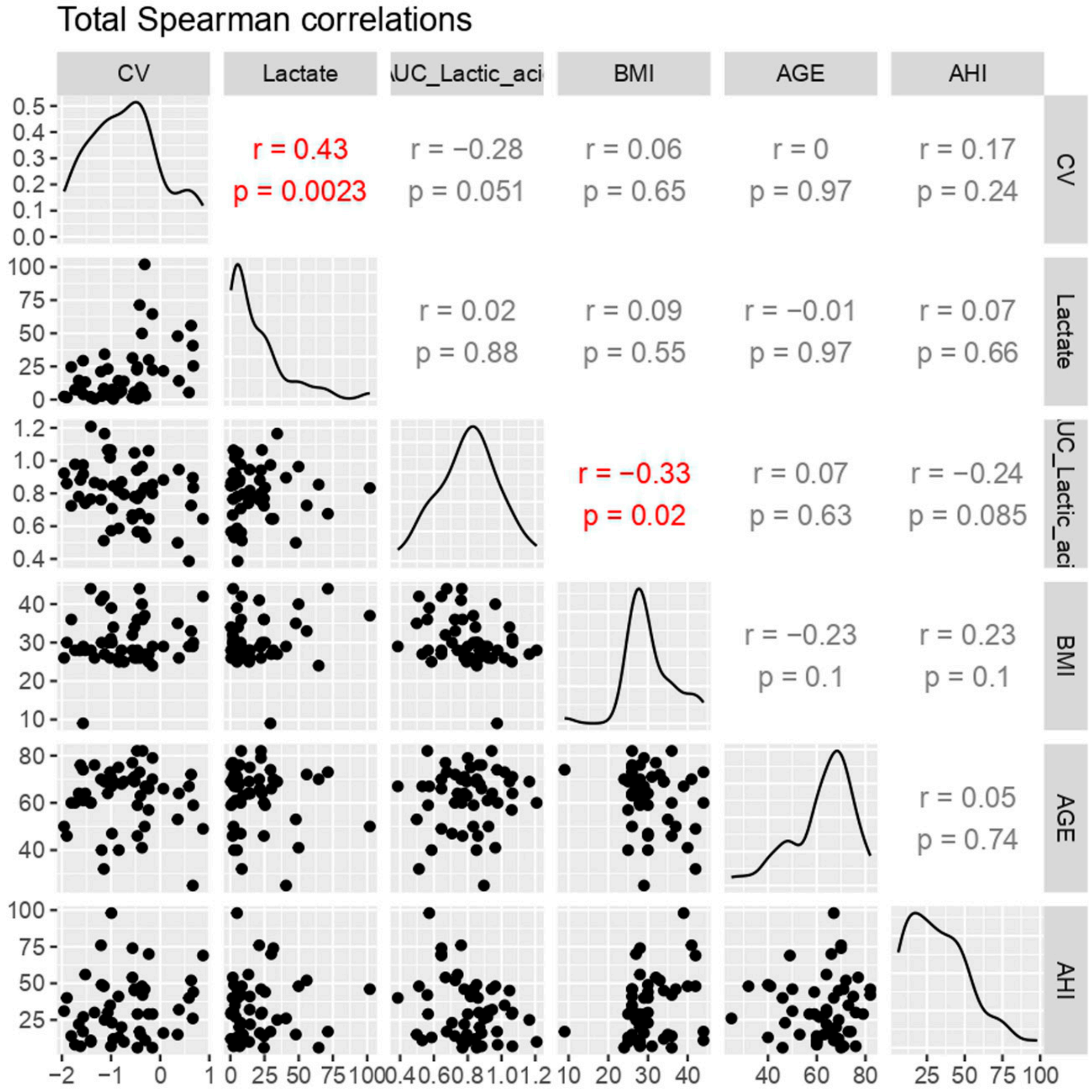

Figure S1. Spearman correlation. A total correlation analysis was performed between the Canonical Variable (CV), the AUC of the lactic acid peak, the lactate concentration, obtained by fluorimetric assay, and clinical data (BMI, age and AHI) using RStudio software. Correlation coefficients (r) and p-value are reported (significant correlations with p-value < 0.05 are highlighted in red).

1) DESCRIPTIVE ANALYSIS

For the first part, the entire dataset of 51 patients was used.

Spearman correlation was employed to analyze the overall correlations between continuous variables, as it is a non-parametric test and robust to outliers.

Significant correlations different from zero were found between lactate and the canonical variable (positive correlation of 0.43, p-value = 0.002, well below 0.05), and between the area under the curve (AUC) of lactic acid and BMI (negative correlation of -0.33, p-value = 0.02). Additionally, the correlation between the AUC of lactic acid and the canonical variable was borderline statistically significant (negative correlation of -0.28, p-value = 0.051).

2) LINEAR REGRESSION MODEL INCLUDING LACTATE AND AUC AS PREDICTORS, WITH CV AS THE DEPENDENT VARIABLE, FOLLOWING OUTLIER REMOVAL

Using the full dataset, a linear model with a quadratic term for lactate was employed to identify outliers and influential values ($CV = \beta_0 + \beta_1 \cdot Lactate + \beta_2 \cdot Lactate^2 + \beta_3 \cdot AUC$). This model was selected based on the information criteria AIC and BIC, which favored it over both the simple linear and cubic models.

After verifying the absence of multicollinearity, normality of residuals, and homoscedasticity, outliers and influential values were identified based on particularly high studentized residuals and large Cook's distances.

Based on these criteria, patients listed in rows 2, 13, 33, 39, 43, and 44 were removed.

Additionally, patient in row 40 was excluded from the analysis due to an exceptionally high lactate value compared to the others, which was considered unreliable.

3) MODEL SELECTION AND INTERPRETATION

The interpretative model was selected using the information criteria AIC and BIC, comparing simple linear, quadratic, and cubic models. The simple linear model was chosen as the best fit:

$$CV = \beta_0 + \beta_1 \cdot Centrated\ Lactate\ + \beta_2\ AUC$$

The coefficient β_1 is 0.018914 with a p-value of 0.00046, indicating a statistically significant positive effect: a one-unit increase in lactate concentration corresponds to an average increase of 0.0189 in CV, holding AUC constant.

The coefficient β_2 is -1.150443 with a p-value of 0.05387, which would be considered statistically significant at an alpha level of 0.10. This indicates that a one-unit increase in the area under the curve (AUC) of lactic acid corresponds to an average decrease of 1.15 in CV, holding lactate concentration constant. Assumptions of absence of multicollinearity, normality of residuals, and homoscedasticity were verified. Effect plots were generated to visualize these relationships

3. RESULTS

3.6 CORRELATION ANALYSIS

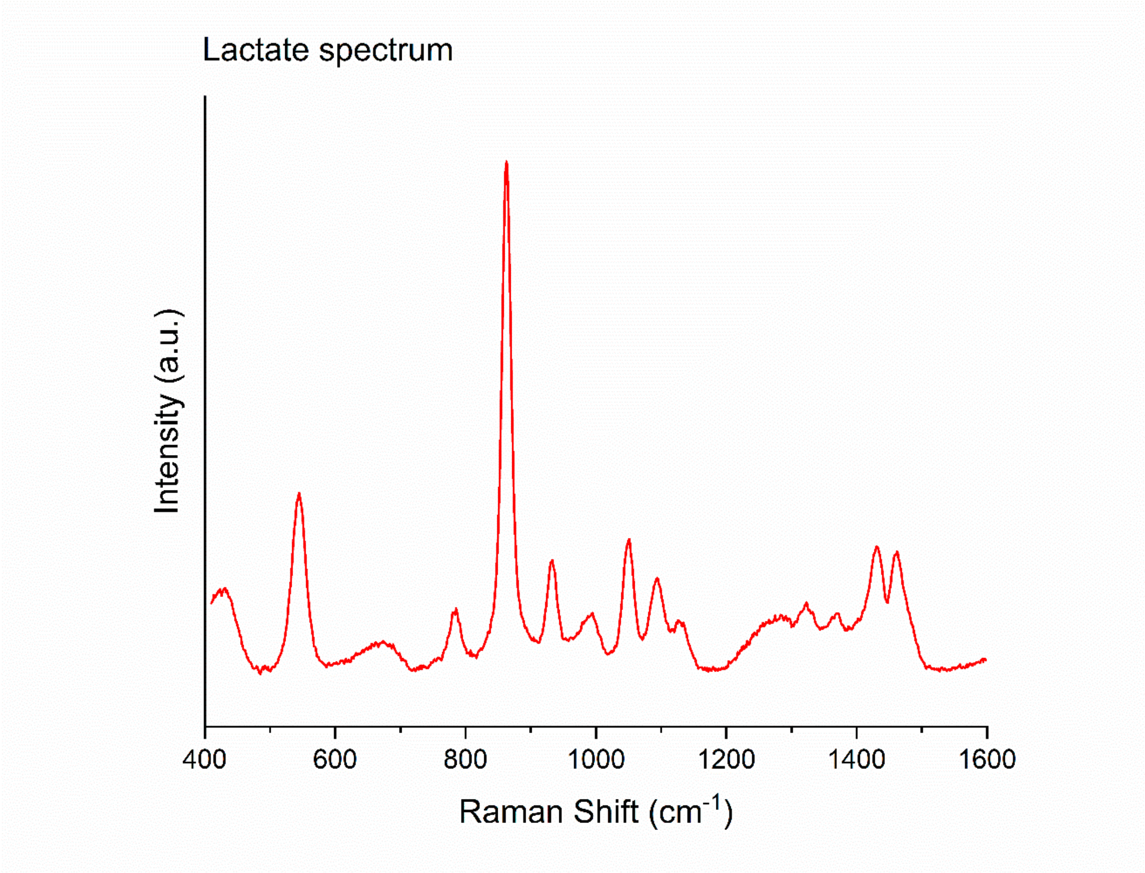

Figure S2. Raman spectrum of Lactate, acquired in the range of 400-1600 cm^{-1}
